# Extirpation of the Spleen

**Published:** 1844-11

**Authors:** 


*Extirpation of the Spleen.*—Dr. Berthet communicated to the Academy of Medicine on the 9th of July, the case of a man who in a quarrel was wounded in the left hypocondrium. Eight days after, when Dr. B. was called to the patient, he found a large tumour formed by the spleen, which was gangrenous; he removed it, and by an appropriate treatment, cured his patient, who lived 13½ years after the accident. Digestion took place as usual, and the individual enjoyed good health. The man ultimately died of pneumonia; at the autopsy, the remains of the spleen were found to be no larger than a filbert, and were adherent to the stomach.

*Med. Times*, July 20.

